# Avoid the fumble around poisonings

**DOI:** 10.4102/phcfm.v2i1.204

**Published:** 2010-08-13

**Authors:** Shabir Moosa

**Affiliations:** 1 Department of Family Medicine, Johannesburg Metro Health District, Gauteng Department of Health, South Africa; 2 Department of Family Medicine, University of Witwatersrand, South Africa

The book *Emergency management of acute poisoning* is a South African title written by Dr Alan Howard, a General Practitioner with an interest in Emergency Medicine. It serves as the prescribed course manual for the Emergency management of acute poisoning (EMAP©) course, further details of which can be found at http://www.emapcourse.co.za.

There are four main sections to this book

- The first is titled ‘General considerations and the approaches to management’ and covers the initial management of an acute poisoning case, including the termination of exposure and the elimination of poison.
- The second section, ‘Poisoning by medication and household agricultural and industrial substances’, provides a detailed list by which one can identify common household and industrial poisonous substances – from analgesics to poisonous plants – with management plans for each.
- The third section, titled ‘Acute poisoning with illicit, addictive substances’, includes identification and management procedures for other illicit and/or addictive substances – from alcohol to opioids – that can induce poisoning.
- The final section, ‘Envenomation in southern Africa’, deals with poisonings resulting from animal bites and/or stings, including those received from various snakes, spiders, scorpions and bees.

*Emergency management of acute poisoning* is a relevant and practical work that addresses how to identify and manage a large variety of common poisonings within our southern African context. It can be a particularly effective tool in this regard when, in the middle of the night in your function as a medical practitioner, for example, you are faced with a causality patient who is in excruciating pain as the result of a swollen leg and you think, half sleepily, ‘Where is that Poison Centre number?’ With this book, though, all the necessary information that can be provided by the Poison Centre is already at your fingertips.

The book is well written and readable, with well-organised contents and prominent headings that enable you to find information in a jiffy. It also has an objective review of case studies at the end of each chapter, so you may study it and jog you memory with that ‘summary’. The appendices are also very useful, providing a list of more ‘memory joggers’ and mnemonics, useful numbers and drug levels, as well as practical skills for use in the initial management.

I do have one criticism of this work, however, which is that it provides too little information on the some of the newer illicit drugs that we might encounter, such as tik in the Western Cape. Nonetheless, this omission is hardly a disqualifier and, as such, I highly recommend this book to medical practitioners who work in primary care emergency settings. To paraphrase Prof. D. Muckart (MD), Head of the King Edward ICU in Durban, South Africa, who wrote the book’s preface – you will not be willing to part with your copy once you have it!

**FIGURE 1:**
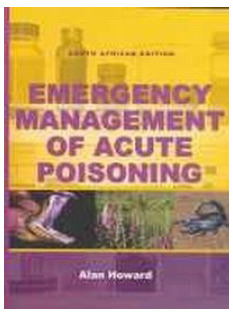
Poisoning of Queen Bona. *Source:* Portrait by Jan Matejkon in 1859. Downloaded from http://en.wikipedia.org/wiki/File:Jan_Matejko-Poisoning_of_Queen_Bona.jpg

